# The Natural History of Untreated Primary Hypogammaglobulinemia in Adults: Implications for the Diagnosis and Treatment of Common Variable Immunodeficiency Disorders (CVID)

**DOI:** 10.3389/fimmu.2019.01541

**Published:** 2019-07-17

**Authors:** Rohan Ameratunga, Yeri Ahn, Richard Steele, See-Tarn Woon

**Affiliations:** ^1^Department of Virology and Immunology, Auckland City Hospital, Auckland, New Zealand; ^2^Department of Clinical Immunology, Auckland City Hospital, Auckland, New Zealand

**Keywords:** CVID, hypogammaglobinaemia, IVIG, intravenous immunoglobulin, SCIG, HGUS

## Abstract

**Background:** Adults with primary hypogammaglobulinemia are frequently encountered by clinicians. Where IgG levels are markedly decreased, most patients are treated with subcutaneous or intravenous immunoglobulin (SCIG/IVIG), because of the presumed risk of severe infections. The natural history of untreated severe asymptomatic hypogammaglobulinemia is thus unknown. Similarly, there are no long-term prospective studies examining the natural history of patients with moderate reductions in IgG.

**Methods:** In 2006, we began a prospective cohort study of patients with symptomatic and asymptomatic reductions in IgG who were not immediately commenced on SCIG/IVIG. Over the course of 12 years, 120 patients were enrolled in the NZ hypogammaglobulinemia study (NZHS) including 59 who were asymptomatic.

**Results:** Five patients with profound primary hypogammaglobulinemia (IgG < 3 g/l), who were not on regular SCIG/IVIG have remained well for a mean duration of 139 months. This study has also shown most asymptomatic patients with moderate hypogammaglobulinemia (IgG 3.0–6.9 g/l) have been in good health for a mean observation period of 96 months. We have only identified one asymptomatic patient with moderate hypogammaglobulinemia who experienced progressive decline in IgG levels to <3 g/l and was accepted for IVIG replacement. Prospective monitoring has shown that none have suffered catastrophic infections or any of the severe autoimmune or inflammatory sequelae associated with Common Variable Immunodeficiency Disorders (CVID). Unexpectedly, 18.1% of asymptomatic and 41.6% of symptomatic hypogammaglobulinemic patients spontaneously increased their IgG into the normal range (≥7.0 g/l) on at least one occasion, which we have termed transient hypogammaglobulinemia of adulthood (THA). In this study, vaccine challenge responses have correlated poorly with symptomatic state and long-term prognosis including subsequent SCIG/IVIG treatment.

**Conclusions:** In spite of our favorable experience, we recommend patients with severe asymptomatic hypogammaglobulinemia are treated with SCIG/IVIG because of the potential risk of severe infections. Patients with moderate asymptomatic hypogammaglobulinemia have a good prognosis. Patients with symptomatic hypogammaglobulinemia are a heterogeneous group where some progress to SCIG/IVIG replacement, while many others spontaneously recover. This study has implications for the diagnosis and treatment of CVID.

## Introduction

Hypogammaglobulinemia is a common clinical scenario. In the majority of severely symptomatic patients with hypogammaglobulinemia, a diagnosis can usually be established and the disorder treated appropriately. Most such symptomatic patients with a defined primary immunodeficiency disorder (PID) are placed on life-long subcutaneous or intravenous immunoglobulin (SCIG/IVIG) therapy.

Evaluation of profound reductions in IgG levels in asymptomatic patients poses a greater challenge. In adults, the principal differential diagnosis is Common Variable Immunodeficiency Disorders (CVID). Patients with untreated CVID are at risk of severe infections including meningitis, sepsis and pneumonia. They are at risk of bronchiectasis and chronic upper respiratory tract disease. A significant minority experience a broad range of autoimmune and inflammatory disorders (1).

The previous ESID/PAGID (1999) CVID diagnostic criteria have been superseded by newer criteria including our (2), ESID registry (2014 and 2019) and CVID ICON (2016) criteria. The ESID registry criteria allow asymptomatic patients to be classified as having CVID if there is a family history and the more recent ICON criteria also allow a diagnosis of asymptomatic CVID providing criteria 2–5 are met (Appendix 1 in Supplementary Material).

Because of concerns about sepsis, asymptomatic patients with severe reductions in IgG levels (<3 g/l) are thus likely to be given a diagnosis of “CVID” and treated with life-long subcutaneous or intravenous (SCIG/IVIG) immunoglobulin. There are no long-term prospective studies of patients with untreated profound asymptomatic hypogammaglobulinemia.

Incidentally discovered milder reductions in IgG can also cause considerable anxiety, as some patients may be at risk of progressing to CVID. Again, there are no cohort studies, which have followed such patients to determine their long-term prognosis.

Standard approaches to patients with hypogammaglobulinemia include a careful history, particularly of infections and autoimmunity, followed by physical examination and laboratory investigations (2). Vaccine challenge responses are commonly undertaken, as a surrogate marker of impaired *in vivo* humoral immunity. Patients are immunized with a panel of vaccines and their antibody responses assessed ~1 month later (3, 4). The previous ESID/PAGID criteria (1999) and the more recent ICON (2016) criteria, place considerable emphasis on impaired responses to vaccine challenges in order to establish a diagnosis of CVID (4–6).

In contrast to other criteria, our 2013 CVID diagnostic criteria require symptomatic disease (Appendix 1 in Supplementary Material) (2, 7). Symptoms resulting from infectious, autoimmune and inflammatory complications are likely to reflect late onset antibody failure (LOAF) leading to immune system failure (ISF). If patients with hypogammaglobulinemia do not meet our criteria for probable CVID, we have classified them as having possible CVID (IgG < 5 g/l) or hypogammaglobulinemia of uncertain significance (IgG 5–6.9 g/l, HGUS) (2). HGUS patients can be either asymptomatic (aHGUS) or symptomatic (sHGUS). Other authors have described similar patients as having IgG deficiency (IgGD), idiopathic primary hypogammaglobulinemia (IPH), unclassified antibody deficiency or unclassified hypogammaglobulinemia (UCH) (8–11). It is common for many such patients to be treated with SCIG/IVIG even though they do not fulfill the criteria for CVID (8, 9).

In 2006, we began a prospective study of patients with symptomatic and asymptomatic primary hypogammaglobulinemia who either declined or did not qualify for SCIG/IVIG, to determine if their long-term outcomes differ. We were particularly interested in the prognosis of asymptomatic patients with mild or severe hypogammaglobulinemia, who were identified during the course of other investigations. Such information would be helpful in making therapeutic decisions and counseling these patients on their long-term prognosis.

This study shows that the majority of asymptomatic patients with hypogammaglobulinemia remain in good health including four with profound hypogammaglobulinemia (IgG < 3.0 g/l), who have not received SCIG/IVIG treatment. Unexpectedly, many patients with symptomatic hypogammaglobulinemia recovered spontaneously. Our findings will be of reassurance to asymptomatic patients with moderate reductions in IgG (3–6.9 g/l). Our findings also have implications for the diagnosis and treatment of CVID.

## Patients and Methods

The primary outcome was recurrent or severe infections leading to SCIG/IVIG replacement. We were especially interested in the prognosis of patients who were offered but declined SCIG/IVIG. Similarly, if other patients began but subsequently discontinued SCIG/IVIG, they were enrolled in the New Zealand hypogammaglobulinemia study (NZHS) to determine the natural history of untreated severe hypogammaglobulinemia.

Another closely related outcome of this study was the stability of IgG levels in asymptomatic patients. This would indicate if they were at risk of developing a disorder such as CVID. Immunoglobulin levels were assessed during clinic visits as well as those ordered by family practitioners, which were linked to electronic records.

The description of the Strobe criteria for this cohort study is as follows: Adult patients (> 16 years) with primary hypogammaglobulinemia (IgG < 7 g/l), attending the private and public practices of the two lead authors (RA and RS) were invited to join the NZHS after informed consent.

Since the main aim of the NZHS was to determine the natural history and prognosis of untreated primary hypogammaglobulinemia, patients were excluded if they commenced SCIG/IVIG within 6 months of assessment. Other exclusion criteria were patients with transient hypogammaglobulinemia of infancy (THI). In order to fulfill criteria for THI, patients must have had a low IgG or globulin level <4 years of age and subsequently made a full recovery (12). Patients who were IgA or IgM deficient, but with normal IgG levels were excluded. Patients with specific antibody deficiency (SAD) who had normal IgG levels but impaired vaccine responses, were also excluded.

At the time of enrolment, each participant's history was assessed by an interviewer-assisted questionnaire. Detailed history was obtained including the reasons for testing, when symptoms began, medication history, hospitalizations, numbers and severity of infections and any treatment received. Particular emphasis was placed on target organ damage (1). Patients were asked to undertake a comprehensive set of laboratory tests as part of their diagnostic evaluation. These included FBC, albumin, serum free light chains, protein electrophoresis, immunophenotype, memory B cells, immunoglobulins, vaccine antibody responses, and isohemagglutinins. Other studies such as fecal calprotectin and fecal alpha 1 antitrypsin were undertaken in selected cases. Given the thorough clinical review and the duration of follow up, it is unlikely secondary causes of hypogammaglobulinemia were undiagnosed (13). Patients were reviewed every 3–6 months depending on the severity of their hypogammaglobulinemia and their symptomatic state. Some patients who had stable IgG levels were followed every 2 years.

All NZ citizens and permanent residents are assigned a unique National Health Index (NHI) number. The NHI is linked to a broad range of health-related investigations and outcomes. The NHI also registers dispensation of pharmaceuticals and blood products including SCIG/IVIG. This has allowed us to review linked computerized hospital medical records and prior laboratory results. Following enrolment, immunoglobulin levels were prospectively measured to determine if any patient experienced progressive deterioration or recovery to normal levels. There was also a retrospective component to this cohort study, in that the total duration of observation was based on the first available immunoglobulin result in linked electronic records.

As part of the standardized approach to primary hypogammaglobulinemia, patients were challenged with diphtheria/tetanus toxoid (DT), H. influenzae type B (HIB), and Pneumovax 23® vaccines (PPV) (14). Post vaccine antibody responses were determined a month later.

Vaccine antibody titers were determined by standard methods. Responses to the Pneumovax 23® were screened with the Binding Site® assay (Birmingham UK), which pools all 23 serotypes. Individual serotype responses were assayed if the Binding Site® result was <270 μg/ml Pneumococcal IgG. An internal audit showed that all patients with a Binding site® result >270 μg/ml achieved > 17/23 Pneumococcal serotypes (>1.3 μg/ml) post vaccination. Similarly if there was no antibody response on the Binding Site® assay (<16 μg/ml), samples were not submitted for serotype specific responses, as the patient was deemed to have failed Pneumovax 23® immunization. Individual pneumococcal serotype responses were measured by the WHO ELIZA (15). Tetanus, diphtheria, and HIB antibodies were measured by ELIZA.

Given its expense, publicly funded SCIG/IVIG treatment is by consensus in Auckland (16). As previously described, patients are offered treatment for severe primary hypogammaglobulinemia with an IgG < 3 g/l (16) regardless of symptomatic state. In the case of symptomatic patients with lesser degrees of hypogammaglobulinemia (IgG 3–6.9 g/l), emphasis is placed on impaired vaccine responses, particularly to Pneumovax 23®. Patients with moderate symptomatic hypogammaglobulinemia are usually placed on prophylactic antibiotics initially before consensus agreement to treat with SCIG/IVIG. Breakthrough infections, in spite of prophylactic antibiotics, are then an indication for SCIG/IVIG treatment in these patients.

## Results

### Patient Demographics and Definitions

**Figure 1:**
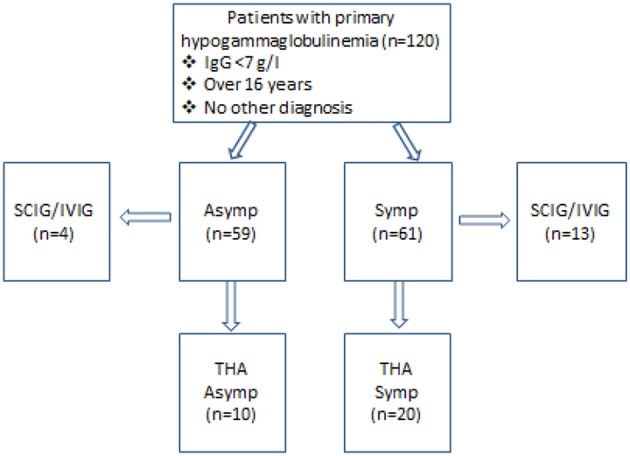
Characteristics of patients enrolled in this study. Fifty-nine patients were asymptomatic while 61 had symptoms, which could be attributed to the immune defect. Thirteen symptomatic (Symp) and four asymptomatic (Asymp) patients were accepted for SCIG/IVIG. THA-transient hypogammaglobulinemia of adulthood, with IgG increasing to ≥7.0 g/l.

Our hypothesis was that symptoms are a reflection of late onset antibody failure leading to immune system failure. We therefore felt symptomatic state would influence consensus decisions to offer SCIG/IVIG treatment. At enrolment, patients were divided according to whether their symptoms could be attributed to the hypogammaglobulinemia (Figure 1). Patients were considered to be asymptomatic if their clinical presentation and reason for testing was unrelated to their hypogammaglobulinemia. Recurrent respiratory tract infections for example were attributed to the hypogammaglobulinemia but not Staphylococcal furunculosis. Patients who had previously been symptomatic but were well in the 2 years preceding enrolment, were classified as being asymptomatic.

The majority of patients were enrolled prior to the advent of the newer CVID criteria and were thus classified purely in terms of symptoms. We found the previous ESID/PAGID 1999 criteria difficult to apply to individual patients.

**Figure 2:**
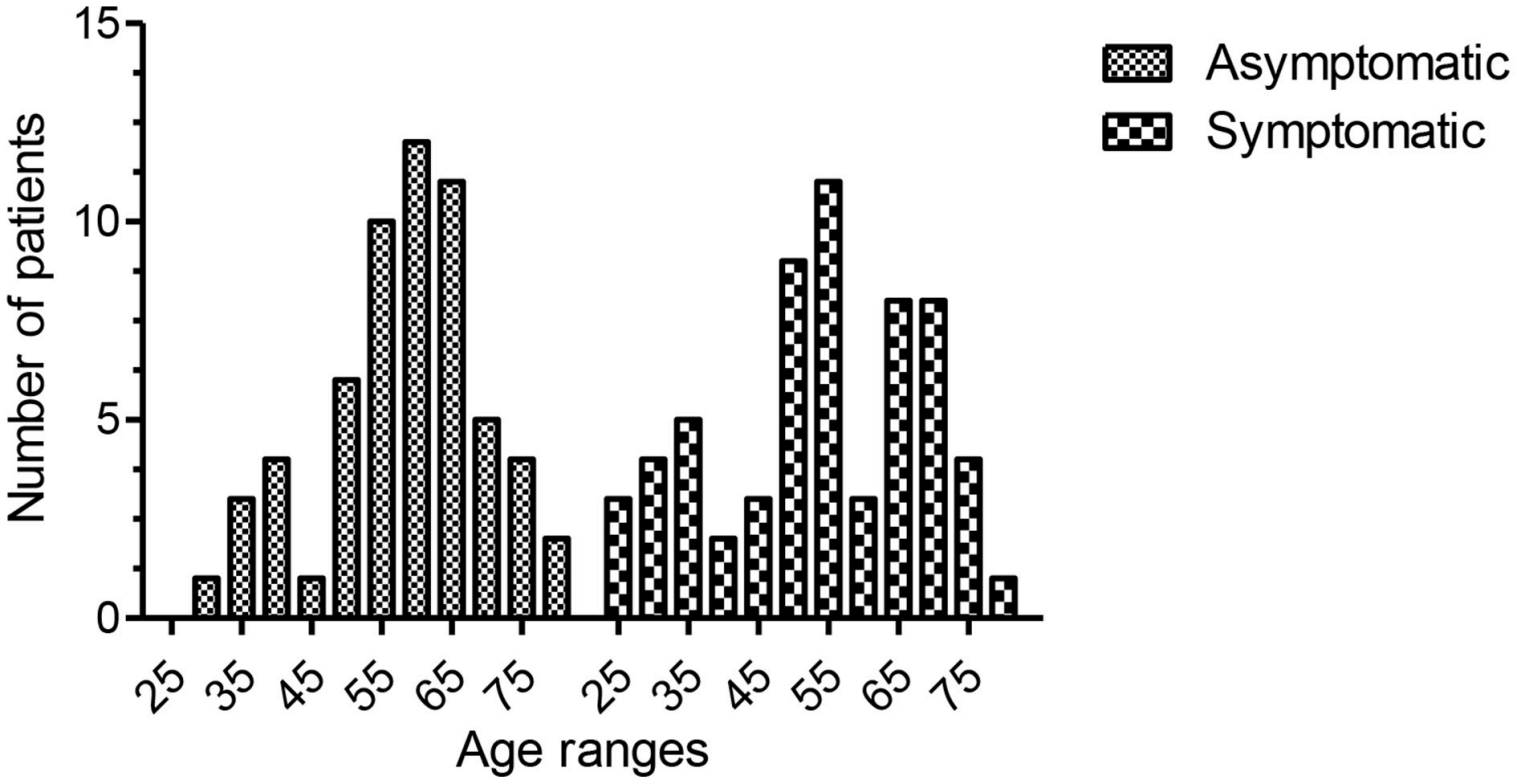
Age ranges of the two groups. The mean age for the entire asymptomatic group was 58.16 (95th CI of mean = 55.18–61.16, range 29–79, sd 11.4, 25th percentile = 50, 75th percentile = 76). The mean age for the entire symptomatic group was 54 (95th CI of mean = 50.2–57.7, range 25–79, sd 14.4, 25th percentile = 45, 75th percentile = 67). There was no statistical difference between the two groups (*p* = 0.079, unpaired *t*-test).

**Figure 3:**
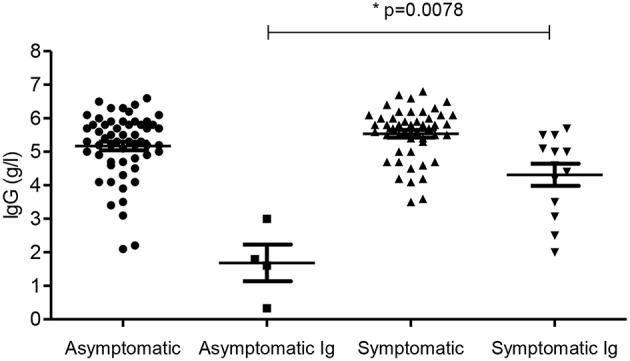
IgG levels in the four groups. Subgroups accepted for SCIG/IVIG are also shown. Ig- patients accepted for SCIG/IVIG. ^*^The asymptomatic Ig group was compared to the symptomatic Ig group by the Mann–Whitney *U*-test.

Fifty-nine patients were asymptomatic while 61 had symptomatic hypogammaglobulinemia (Figure 1). Demographic details are presented in Table 1 and age ranges are shown in Figure 2. The lowest immunoglobulin results from linked electronic records are shown in Figure 3. Of individuals not accepted for SCIG/IVIG (Table 1), the asymptomatic group had a significantly lower IgG (5.17 g/l) than the symptomatic group (IgG 5.54 g/l, *p* = 0.0388 Mann–Whitney *U*-test). The mean duration of observation was 96 months in the asymptomatic group and 85.1 months in the symptomatic group, which was similar (*p* = 0.234, unpaired *t*-test).

**Table 1:** Clinical and laboratory characteristics of the patients in this study.

**Clinical features**	**Asymptomatic *n* = 55**	**Asymptomatic (SCIG/IVIG) *n* = 4**	**Symptomatic *n* = 48**	**Symptomatic (SCIG/IVIG) *n* = 13**
Male/Female	20/35	2/2	10/38	4/9
Mean age	58	60	54.4	52.6
Family history of PID	6	1	7	2
Observation period (months)	96	55^*^	85.1	75.7^*^
Meningitis/sepsis	0/0	0/0	2/2	1/3
Chronic sinus disease	2	0	29	7
Mastoiditis	0	0	0	0
GLILD	0	1	0	2
Bronchiectasis	0	0	12	8
Pneumonia	2	0	8	5
Asthma	11	0	21	6
Severe viral infections	0	0	0	0
Severe autoimmunity	0	0	0	1
Enteritis/colitis^**^	0	0	0	1
Liver disease	0	1 (cholecystitis)	0	0
Malignancy^***^	4	0	3	3
Cytopenias	1	1	0	1
Prophylactic antibiotics	3	0	15	4

*SCIG/IVIG, subcutaneous or intravenous immunoglobulin; NRH, Nodular Regenerative Hyperplasia; Dip, Diphtheria; Tet, Tetanus; HIB, Haemophilus influenzae type b; GLILD, granulomatous-lymphocytic interstitial lung disease; IT, immunotherapy*.

* Mean time to treatment with SCIG/IVIG,

** One patient had inflammatory bowel disease (Crohn's),

*** *Malignancy includes ca breast, uterus, ca cervix and melanoma*.

In the asymptomatic group, 8 were identified as having reduced globulins during testing for urticaria, 2 for other rashes, 1 for asthma, 1 for fatigue, 2 during clinical research studies, and remainder (*n* = 45) during routine blood tests by their family physician. The low globulin results triggered immunoglobulin assessment. In contrast, the majority of the symptomatic group had immunoglobulin tests during evaluation of infections (*n* = 52). The remainder were tested for a variety of reasons including immune thrombocytopenia, fatigue and abdominal symptoms.

### Treatment With SCIG/IVIG

**Figure 4:**
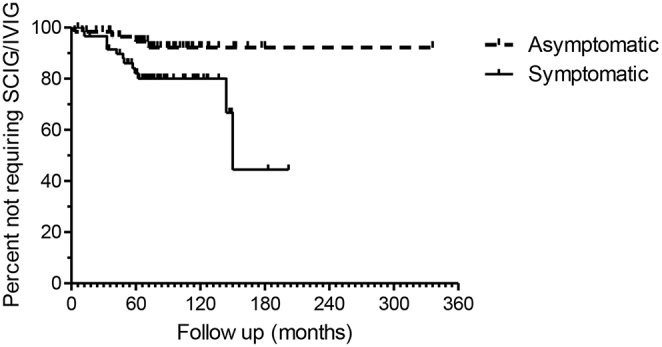
Kaplan–Meier plots of the asymptomatic and symptomatic patients. The endpoint is time to SCIG/IVIG treatment. The duration of observation is based on the first available IgG level in NHI-linked electronic records. The difference in the two survival curves is highly significant (*p* = 0.0147), Log rank Mantel Cox test.

Four patients (6.6%) in the asymptomatic group were offered SCIG/IVIG compared to 13 (21.3%) in the symptomatic group (Figure 4), a significant difference (*p* = 0.022, Chi square test). The IgG was much lower in the asymptomatic group vs. the symptomatic group who were offered SCIG/IVIG (*p* = 0. 0078 Mann–Whitney *U*-test) (14).

Two asymptomatic and one symptomatic patient with severe hypogammaglobulinemia declined regular SCIG/IVIG therapy. The two asymptomatic patients declining or discontinuing regular SCIG/IVIG had profound hypogammaglobulinemia (IgG 1.6 and <0.33 g/l). Two other asymptomatic patients with IgG levels <3 g/l were not offered SCIG/IVIG because of excellent health. A fifth asymptomatic patient (IgG 1.8 g/l), who initially declined IVIG, accepted the need for treatment after CT scans showed the presence of granulomatous lymphocytic interstitial lung disease (GLILD). A sixth asymptomatic patient was accepted for treatment after progressive decline in her IgG to <3 g/l.

**Figure 5:**
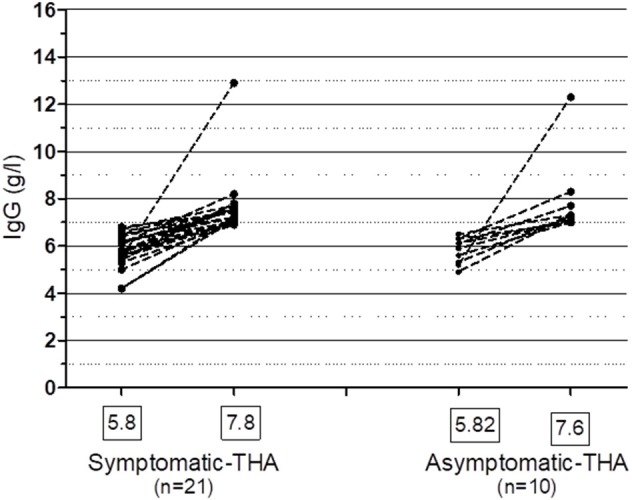
Spontaneous recovery of IgG ≥ 7.0 g/l in patients with symptomatic and asymptomatic hypogammaglobulinemia on at least one occasion. One patient who was treated with IVIG, who recovered her IgG is included in the Symptomatic group. THA, transient hypogammaglobulinemia of adulthood. Mean IgG levels (g/l) are shown in the boxes.

The symptomatic patient declining SCIG/IVIG (IgG 2.5 g/l) had recurrent chest infections and mild GLILD, which has not progressed in the last 5 years. Another three symptomatic patients subsequently discontinued IVIG and in one there was improvement in the IgG levels (4.2 g/l) into the normal range (7.1 g/l). This patient was also included in Figure 5. The remaining 9 symptomatic patients have continued SCIG/IVIG long-term.

### Prophylactic Antibiotics

Three of 59 (5%) asymptomatic patients received prophylactic antibiotics vs. 19 of 61 patients (31%) in the sHGUS group (*p* = 0.000225, Chi square test). Within the asymptomatic group, one patient was given prophylactic antibiotics for urinary tract infections (UTI), another for Staphylococcal furunculosis and one had previously needed antibiotic prophylaxis for respiratory infections. She was classified as asymptomatic because she had stopped antibiotics 2 years prior to enrolment. The 19 patients in the symptomatic group received prophylactic antibiotics for recurrent respiratory tract infections.

### Laboratory Tests Including Vaccination Studies

Nine of fifty five patients in the asymptomatic group (16.3%) had reduced IgA levels (<0.7 g/l) but none were IgA deficient (<0.07 g/l). Three of four patients (75%) in the asymptomatic group who were offered SCIG/IVIG, had reduced IgA levels. There were 13 (27%) patients with reduced IgA levels including two with IgA deficiency in the symptomatic group. Five of 13 symptomatic patients treated with SCIG/IVIG had reduced IgA levels including two who were IgA deficient. Ten (18.2%) asymptomatic patients had reduced IgM (<0.4 g/l) levels compared to five (10.4%) with symptoms.

Switched memory B cells were reduced in both groups and did not distinguish those who were subsequently accepted for SCIG/IVIG replacement (Table 1). We have previously shown intra-individual variability in memory B cell assays in CVID patients but these were not repeated (17).

The majority of both asymptomatic (43/59) and symptomatic (54/61) patients underwent vaccine challenge responses (Table 2). We have presented both pre-vaccination protective levels as well as the higher threshold levels we have published in our CVID diagnostic criteria (Table 2). The higher levels are derived from studies of normal individuals undergoing booster vaccination in the community (2). We have also shown the numbers of patients experiencing a x4 increase in antibody levels and those achieving the higher threshold levels following vaccination. Some patients had high baseline antibody levels at evaluation and were not vaccinated to that specific vaccine. Some patients declined vaccination, so only their baseline antibody results are available. Others did not undertake post-vaccination blood testing. The denominators reflect this incomplete data.

**Table 2:** Laboratory results including vaccine responses.

**Laboratory results**	**Asymptomatic *n* = 55**	**Asymptomatic SCIG/IVIG *n* = 4**	**Symptomatic *n* = 48**	**Symptomatic SCSIG/IVIG *n* = 13**
IgG (g/l)	5.17	2.1	5.54	4.3
IgA (g/l) <0.7/ <0.07 g/l	9/0	3/0	13/2	5/2
IgM (g/l) <0.4	10	2	5	2
THA (IgG ≥ 7 g/l)	10	0	20	1
Reduced switched memory B cells (CD19+, CD27+, IgD−)	16/47	3/4	16/44	5/11
Vaccine challenge responses	40/55	3/4	41/48	13/13
Dip > 0.1 IU/ml	38/54	0/4	38/48	11/13
Dip > 1 IU /ml	8/54	0/4	12/48	5/13
^*^Dip post vac x4	16/38	0/3	14/37	4/13
^*^Dip post vac > 1 IU/ml	6/38	0/3	12/37	5/13
Tet > 0.1 IU/ml	52/52	3/4	47/47	13/13
Tet > 1 IU/ml	45/52	2/4	43/47	12/13
^*^Tet post vac x4	23/39^*^	2/3^*^	21/39^*^	4/12^*^
^*^Tet post vac > 1 IU/ml	37/39	2/3	39/39	11/12
HIB > 0.15 μg/ml	49/49	3/4	43/43	12/12
HIB > 1 μg/ml	36/49	2/4	36/43	11/12
^*^HIB post vac x4	19/33^*^	1/2^*^	20/30^*^	4/8^*^
^*^HIB post vac > 1 μg/ml	31/33	2/2	29/30	8/8
Pneumovax®				
Binding site > 16 μg/ml	43/50	2/4	43/46	10/12
^**^Serotypes > 17/23	9/33^***^	0/2	10/25	1/8^***^
Isohemagglutinins > 1:2	47/48	4/4	42/42	8/12

*Vaccine responses are presented as those achieving protective levels and the higher levels we have advocated for our CVID criteria*.

* *Below are patients who underwent booster vaccination, those achieving a x4 increase from baseline and those achieving the higher levels we have advocated for our CVID criteria. Note that patients with high baseline levels were not able to achieve an x4 increase following booster immunization because of the assay measurement limits. This underestimates vaccine responses to these antigens*.

** *All patients were vaccinated with Pneumovax 23®*.

*** *There was no significant difference (p = 0.382, Chi square test) in Pneumovax 23® responses between asymptomatic patients and symptomatic patients accepted for SCIG/IVIG. Not all patients returned for post vaccine bloods. The denominator reflects these incomplete results. Dip, diphtheria antibodies; HIB, H. influenzae type B antibodies; Tet, tetanus antibodies*.

### Application of Newer Diagnostic Criteria for CVID

We have used clinical and laboratory data to classify patients according to the three newer sets of CVID criteria (Table 3) described in Appendix 1 (Supplementary Material). Each patient received three separate diagnoses based on currently used criteria for CVID. Given the difficulties interpreting the ESID/PAGID 1999 criteria, these were not considered in this study (2).

**Table 3:** Retrospective application of three commonly used criteria for CVID.

**Diagnostic criteria**	**Asymptomatic *n* = 45**	**Asymptomatic SCIG/IVIG *n* = 4**	**Asymptomatic THA *n* = 10**	**Symptomatic *n* = 28**	**Symptomatic SCIG/IVIG *n* = 13**	**Symptomatic THA *n* = 20**
**Ameratunga**						
AHGUS	29	0	9	0	0	0
SHGUS	0	0	0	19	6	18
PCVID	16	4*	1	6	2	2
CVID	0	0	0	3	5	0
**ESID**						
UCH	41	2	10	22	9	20
CVID	4	2	0	6	4	0
**ICON**						
UCH/IgGD	41	1	10	22	4	20
CVID	4	3	0	6	9	0

The majority of patients were enrolled before these criteria were described. THA patients were assessed during the period of hypogammaglobulinemia. *One patient had asymptomatic GLILD (see text for further discussion of classification). See Figure 1 for the cohort study characteristics and Appendix 1 in Supplementary Material for the relevant definitions of the criteria. AHGUS, asymptomatic hypogammaglobulinemia of uncertain significance; SHGUS, symptomatic hypogammaglobulinemia of uncertain significance; THA, transient hypogammaglobulinemia of adulthood; UCH, unclassified antibody deficiency or unclassified hypogammaglobulinemia; IgGD, IgG deficiency; PCVID, possible CVID.

There were no patients fulfilling criteria for late onset combined immunodeficiency (LOCID) in this cohort. Although the ESID and ICON CVID criteria exclude such patients, we have argued these patients fall within the broad spectrum of CVID and CVID-like disorders (18). They remain within the Ameratunga et al. CVID criteria.

### Transient Hypogammaglobulinemia of Adulthood

Of patients not placed on SCIG/IVIG, significantly more symptomatic patients (20/48, 41.6%) than asymptomatic patients (10/55, 18.1%) spontaneously normalized their IgG levels to ≥7.0 g/l on at least one occasion (*p* = 0.006, Chi square test). The lowest and highest immunoglobulin results in these patients are shown in Figure 5. There was fluctuation of IgG levels around the lower reference interval after normalization; five patients in the asymptomatic and 9 symptomatic patients subsequently had one or more IgG levels below 7 g/l. The immunoglobulins were confirmed as being in the normal range on average of 2.1 times in the group with sustained normalization of IgG. Although the average improvement in IgG was ~2 g/l in both groups, several patients experienced greater increases (Figure 5). We have termed these patients transient hypogammaglobulinemia of adulthood (THA, Figure 1). Many THA patients chose not to return for follow-up clinic visits, as they felt they were “cured.”

Twenty patients with symptomatic hypogammaglobulinemia had bronchiectasis (Table 1). There was no other explanation for the bronchiectasis in these patients. Of the 20, eight were accepted for SCIG/IVIG replacement. The other 12 were declined by consensus, either on the basis of satisfactory vaccine responses (16) or because of clinical improvement with prophylactic antibiotics. Their lowest IgG levels varied from 5.0 to 6.5g/l. Of the 12 bronchiectasis patients not accepted for SCIG/IVIG, seven (58.3%) spontaneously normalized (≥7.0 g/l) their IgG levels on at least one occasion.

### Underlying Causes of Hypogammaglobulinemia

Family history of hypogammaglobulinemia was slightly more common in patients with symptoms (14.7%) compared with asymptomatic patients (10%), which was not statistically significant (*P* = 0.67, Chi square test). We did not have consent to access to the relative's notes and cannot be more precise about their diagnoses.

One of the asymptomatic patients declining SCIG/IVIG had genetic studies and was shown to be homozygous for the TACI C104R mutation (19–21). He was part of a family where we demonstrated the existence of quantitative epistasis in humans as a result of digenic inheritance (22). Whole exome sequencing was undertaken in two families. Patients with causative mutations for CVID-like disorders were excluded from this study (18, 22, 23).

Similarly, several patients with secondary causes for hypogammaglobulinemia were identified but were also excluded from this study. These included anticonvulsant medications (24, 25), a patient with undiagnosed celiac disease (26), one with Munchausen syndrome (27), one on prednisone and some with lymphoproliferative disorders.

### Long Term Clinical Outcomes

One asymptomatic patient underwent laparoscopic cholecystectomy for presumed unrelated cholelithiasis. None of the asymptomatic patients developed recurrent infections or severe autoimmunity requiring SCIG/IVIG. Four asymptomatic patients had malignancy; two ca breast, one ca uterus and one melanoma, which were not attributed to the hypogammaglobulinemia. None of the patients in this study developed ca stomach or lymphoma, which have been more closely linked to immunodeficiency.

Two asymptomatic patients were found to have mild chronic sinus disease during evaluation of their hypogammaglobulinemia. In contrast, 36/61 symptomatic patients had chronic sinus disease. The majority underwent surgery and were clinically improved. One symptomatic patient improved after tonsillectomy.

One symptomatic patient had Crohn's disease and another had chronic diarrhea but endoscopy did not show nodular lymphoid hyperplasia (NLH) of the gut. Two symptomatic patients treated with IVIG had GLILD, and one in addition had cytopenias, sequelae typically associated with CVID (Table 1). None of the patients died during the study.

## Discussion

This long-term prospective cohort study has given us insight into the natural history of asymptomatic and symptomatic primary hypogammaglobulinemia in older adults. A prospective study reduces the risk of recall bias. The standardized interviewer-assisted questionnaire, review of electronic notes and linked laboratory results further enhanced the quality of data. There are however limitations to this study. Some patients did not undertake routine blood tests after enrolling in the study, while others declined vaccine challenges or did not undertake follow up bloods. The denominators in Table 1 have been adjusted to accommodate these incomplete results.

Many patients with THA chose to discontinue follow up and of those not treated with SCIG/IVIG, 5/45 asymptomatic and 3/27 symptomatic patients have not had contact since 2015. They were assumed to be lost to specialist follow up. However, because of NHI numbers and linked computer records, we are confident we were able to identify all patients who subsequently received SCIG/IVIG, which was the main outcome of the study. Review of electronic notes has confirmed none of the patients lost to follow up have required hospital admission for severe infections or autoimmunity.

Many more symptomatic patients (13/61) were accepted for SCIG/IVIG treatment than those without symptoms (4/59) supporting our hypothesis that symptoms may change perceptions of the severity of hypogammaglobulinemia. Asymptomatic patients accepted for SCIG/IVIG, had much lower levels of IgG compared to symptomatic patients offered SCIG/IVIG (Figure 3), further supporting this hypothesis. Asymptomatic patients with profound hypogammaglobulinemia (mean = 55 months) were accepted for SCIG/IVIG sooner than symptomatic patients (mean = 75.7 months) with moderate hypogammaglobulinemia (Table 1). Asymptomatic patients with profound hypogammaglobulinemia are not usually required to trial prophylactic antibiotics before being accepted for SCIG/IVIG treatment, which may account for this temporal difference.

Five patients with severe hypogammaglobulinemia (IgG < 3 g/l) including three who declined long-term SCIG/IVIG treatment have remained well for a mean observation period of 139 months. Apart from the patient with coincidental cholecystitis from cholelithiasis leading to laparoscopic cholecystectomy, there were no severe infections during the observation period. Patients declining SCIG/IVIG treatment have been made aware of the risks of sepsis, meningitis and pneumonia. The precise risk is however difficult to quantify. This has medico-legal implications and has been carefully discussed with these patients. We have recommended a very low threshold for seeking medical advice and have advised these patients to carry home antibiotics.

We have classified the patient with profound hypogammaglobulinemia (IgG 1.8 g/l) who had no symptoms at presentation, but was subsequently found to have GLILD, as being asymptomatic (Table 3). Her clinical presentation was unrelated to her profound hypogammaglobulinemia or GLILD. We acknowledge this patient could have been considered to have a “CVID-defining” lesion, in the context of severe primary hypogammaglobulinemia and to have met our CVID criteria. A similar scenario could apply to an asymptomatic patient coincidentally found to have severe cytopenias and reduced IgG levels.

This patient's asymptomatic GLILD improved after commencing IVIG. She has not needed additional immunosuppression. This demonstrates CT scans of the thorax and sinuses should be routinely undertaken in all patients with severe hypogammaglobulinemia. It also illustrates the heterogeneity of GLILD as one symptomatic patient with the disorder, declining SCIG/IVIG, has remained stable with no treatment. He continues to be closely monitored.

In spite of our favorable experience, we strongly recommend patients with severe asymptomatic hypogammaglobulinemia undergo treatment with SCIG/IVIG because of the potential risk of severe infections. With the current global measles outbreak, these patients have been advised to receive immune globulin if there is a known exposure or to have regular SCIG/IVIG even on a temporary basis.

Some of these asymptomatic patients with profound hypogammaglobulinemia could become symptomatic following an environmental trigger such as a viral infection, later in life. It is known that some patients with CVID can develop symptoms for the first time in their 7th or 8th decade. Herpes virus infections are being increasingly recognized as a trigger for lymphoproliferative disease in some patients with CVID-like disorders (21). This is analogous to patients with X-linked lymphoproliferative disease, where the disorder can be triggered by an EBV infection (28).

It is apparent from this study that the majority patients with moderate asymptomatic hypogammaglobulinemia (IgG 3.0–6.9 g/l) also remain well for many years. There have been no apparent severe infective, autoimmune or inflammatory sequelae requiring SCIG/IVIG during the period of follow up. We have only identified one of 59 asymptomatic patients who experienced gradual decline in IgG levels culminating in IVIG replacement. Most asymptomatic patients with hypogammaglobulinemia have not experienced deterioration of their IgG below 3 g/l. This observation will be of considerable reassurance to patients with moderate asymptomatic hypogammaglobulinemia.

It is likely many more patients with asymptomatic hypogammaglobulinemia will be identified in the future with screening of globulin results and reflex testing of IgG (29). It will be important for such asymptomatic patients to be closely followed to confirm they have a good long-term prognosis.

At this time we have not undertaken genetic studies in the majority of these patients. It is possible some carry mutations of genes predisposing to CVID (TACI, BAFFR, MSH5, TWEAK) or causing (NFKB1, NFKB2, etc.) CVID-like disorders. We and others have shown that some patients carrying mutations for CVID-like disorders can be asymptomatic (18). Our patient with the homozygous C104R mutation of the TACI gene has not experienced an increased number or severity of infections for over a decade in spite of an IgG of 1.6 g/l (22). Again it is possible a viral infection could trigger symptomatic disease in such patients with a genetic predisposition to CVID and alter their prognostic trajectory.

This study has fiscal implications for funders of SCIG/IVIG. The life-time cost for SCIG/IVIG based on adjusted body weight and maintenance according to trough IgG levels (30) is approximately $1–2M per patient. Our study indicates asymptomatic patients with moderate hypogammaglobulinemia remain well and may not require immunoglobulin replacement. They do however require regular follow up as there is a small but significant possibility their IgG levels will progressively decline and they will need SCIG/IVIG, as seen in one patient in this study.

While we anticipated more symptomatic patients would require SCIG/IVIG, we did not expect 41.6% to spontaneously recover their IgG levels into the normal range on at least one occasion. Most experienced sustained increases in their IgG. There were no obvious secondary causes identified in this group.

Immunoglobulin assays in the Auckland region are undertaken by four laboratories. Results are standardized through sample exchanges via the Auckland Regional Quality Assurance Group (ARQAG) to minimize inter-laboratory variation. All laboratories participate in the Royal College of Pathologists of Australasia (RCPA) external quality assurance program (QAP), which allows comparison of various platforms measuring IgG levels. The intra-laboratory co-efficient of variation (CV) of immunoglobulin assays by turbidimetry (Cobas), which is a measure of precision, is <1%. Inter-laboratory IgG assays are more variable. We have ascertained where each assay was performed on linked computerized laboratory results. Only one patient in the asymptomatic and two in the symptomatic group had assays in different laboratories, which might account for variable IgG results.

Thus, neither patient factors nor inter-laboratory imprecision can account for the recovery of IgG in most of these patients. Transient hypogammaglobulinemia of adulthood is an appropriate term for this group and is analogous to THI, although the underlying immunological mechanisms are likely to differ between adults and children. We have shown patients with THI can recover in their second or third decade (12). Older adults with THA are likely to differ from children and young adults recovering from THI.

This study will complicate the immunological assessment of bronchiectasis as the majority of patients not receiving SCIG/IVIG recovered their IgG levels ≥7.0 g/l on at least one occasion. It is possible IgG levels fluctuate during infective exacerbations of bronchiectasis. This study was not designed to examine this hypothesis, which could be considered in future prospective studies of patients with bronchiectasis and hypogammaglobulinemia.

Our observations have reinforced the importance of measuring IgG levels on several occasions, particularly if there is no immediate need to commence SCIG/IVIG. Multiple abnormal immunoglobulin results from the same laboratory will make decisions about life-long SCIG/IVIG treatment more robust. This is illustrated by one patient discontinuing IVIG who normalized her IgG. Although standard clinical practice mandates repeating reduced immunoglobulin levels, the ESID registry and the recent ICON criteria for CVID have formalized this (5). Our study supports this recommendation.

This variability in IgG levels will also complicate application of most diagnostic criteria for CVID. The threshold for the hypogammaglobulinemia has been set at 2 sd below the mean (IgG < 7 g/l) for most CVID diagnostic criteria (6). As seen here, there is considerable intra-individual variability of IgG levels around this threshold. We have set our CVID threshold of IgG at 5 g/l (2). At this level only one asymptomatic and two symptomatic patients recovered their IgG levels to >7.0 g/l would be classified as THA. This variability is a strong argument for revising the IgG cut-off in other CVID diagnostic criteria. We feel HGUS is an appropriate term for these patients as it reflects variability both in clinical course and IgG levels.

We have applied commonly used CVID diagnostic criteria to this cohort of patients (Table 3). Since the majority of patients were enrolled before these newer criteria were described, this cohort could be considered relatively unbiased as decisions to offer treatment were not influenced by diagnosis in the majority.

The CVID criteria are shown in Appendix 1 (Supplementary Material). As seen in Table 3, there is congruence in the majority of patients. Important differences are however noted. The Ameratunga et al. (2) CVID criteria require symptomatic disease, thus asymptomatic patients with an IgG < 5 g/l will be classified as having possible CVID. A reduction in IgA and/or IgM is not mandatory in the Ameratunga et al criteria for a diagnosis of probable CVID, although these are in the category C criteria (Appendix 1 in Supplementary Material). The ESID (2014) criteria in contrast require a reduction in IgA, while the ICON criteria require a reduction in IgA and/or IgM to establish a diagnosis of CVID (4, 11). Thus, any patient with low IgG levels but normal IgA and IgM will be classified as UCH or IgGD by the latter criteria.

The asymptomatic patient with absent IgG (<0.33 g/l) for over two decades has normal IgA and IgM levels and would not be classified as CVID by ESID or ICON criteria. Since relevant symptoms are a prerequisite for our CVID diagnostic criteria (Appendix 1 in Supplementary Material) (2, 5, 7) this patient would be termed possible CVID by the Ameratunga et al. criteria. He would nevertheless qualify for SCIG/IVIG, as our CVID criteria state clinical judgement is paramount in managing these complex patients (2).

This study illustrates the difficulties in undertaking and interpreting vaccine challenge responses in patients with hypogammaglobulinemia. We have documented vaccine responses in both asymptomatic and symptomatic patients. As predicted, pneumococcal serotype responses were reduced in symptomatic patients offered SCIG/IVIG, which reflects the consensus approach to offering treatment. Pneumococcal vaccine responses were however also impaired in other groups including asymptomatic individuals (Table 2). There was no statistical difference in impaired pneumococcal vaccine responses between symptomatic patients accepted for SCIG/IVIG vs. asymptomatic patients with mild hypogammaglobulinemia (*p* = 0.382, Chi square test). This illustrates the poor specificity of using pneumococcal vaccine responses to make decisions about SCIG/IVIG treatment.

The need for vaccination responses differs between these CVID criteria. In contrast to the ICON criteria, it is possible to make a diagnosis of CVID without impaired vaccination responses in the Ameratunga et al and ESID registry criteria (Appendix 1 in Supplementary Material). We have not attempted to correlate vaccine responses with CVID diagnostic criteria as the ICON criteria applied here require poor vaccine responses. There is thus a risk of fallacious circular logic. It is however apparent that diphtheria and pneumococcal responses were impaired in otherwise healthy asymptomatic persons with mild hypogammaglobulinemia, who are unlikely to have CVID or any other immunological disorder (Table 2). Similarly, the excellent tetanus and HIB responses in symptomatic patients were non-discriminatory. This study reinforces our concerns about the utility and interpretation of vaccine responses to diagnose CVID or to determine eligibility for SCIG/IVIG (6).

Some studies have however shown impaired vaccine responses have correlated more closely with symptomatic state (31, 32). In one study, responses to tick-borne encephalitis vaccine was used as a neoantigen, responses which segregated with symptomatic state (31). The typhoid, ϕx174 and rabies vaccines have also been used as neoantigens (3, 33). Such an approach avoids preserved secondary antibody responses by memory B cells from primary immunization series. Currently, the use of these vaccines as neoantigens is not standard practice. There are concerns about the safety of the rabies vaccine (in this context) and the ϕx174 vaccine is not registered (6). It is possible the use of neoantigens may have shown more effective separation of patient groups described in this study.

Here, we have shown that the majority of asymptomatic patients with moderate hypogammaglobulinemia (IgG 3.0–6.9 g/l) remain in excellent health and the risk of deterioration is very low. In contrast, those with symptoms are a heterogeneous group; although many more were offered SCIG/IVIG, more than one third recovered their IgG into the normal range on at least one occasion. Patients with symptomatic hypogammaglobulinemia thus need closer follow up, as their prognosis is more variable.

This prospective cohort study has demonstrated that symptoms rather than vaccine responses predict long-term prognosis in patients with primary hypogammaglobulinemia. This is consistent with our hypothesis that symptoms reflect immune system failure caused by late onset antibody failure. Our study illustrates the complexity of patients presenting with primary hypogammaglobulinemia, the need for thorough clinical assessment and long-term follow up by experienced clinical immunologists (2).

## Data Availability

The datasets for this study will not be made publicly available because of confidential patient clinical information, which may allow identification of individuals.

## Ethics Statement

This study was approved by the Health and Disability Ethics Committee of the NZ Ministry of Health (HDEC) and the ADHB ethics committee (3435). Written informed consent was obtained from all participants.

## Author Contributions

All authors listed have made a substantial, direct and intellectual contribution to the work, and approved it for publication.

### Conflict of Interest Statement

The authors declare that the research was conducted in the absence of any commercial or financial relationships that could be construed as a potential conflict of interest.
