# Supplementary material for: The Natural History of Untreated Primary Hypogammaglobulinemia in Adults: Implications for the Diagnosis and Treatment of Common Variable Immunodeficiency Disorders (CVID)
Source: Front Immunol. 2019 Jul 17;10:1541. doi: 10.3389/fimmu.2019.01541 (PMC6652801; doi:10.3389/fimmu.2019.01541)
Supplement: Supplementary file 1 [file Data_Sheet_1.docx]

**Appendix 1. Current diagnostic criteria for CVID**. These criteria are copied verbatim from the original publications to ensure accuracy. The original sources are acknowledged.

**Ameratunga et al criteria 2013^1^**

| **A** | Must meet all major criteria |
| --- | --- |
|  | - Hypogammaglobulinemia IgG < 5 g/l. ^2^ - No other cause identified for immune defect. ^3^ - Age > 4 years. ^4^ |
| **B** | Sequelae directly attributable to immune system failure (ISF) (1 or more) |
|  | - Recurrent, severe or unusual infections. - Poor response to antibiotics - Breakthrough infections in spite of prophylactic antibiotics - Infections in spite of appropriate vaccination eg HPV disease - Bronchiectasis and/ or chronic sinus disease - Inflammatory disorders or autoimmunity. ^5^ |
| **C** | Supportive laboratory evidence (3 or more criteria) |
|  | - Concomitant reduction or deficiency of IgA (< 0.8 g/l) and/or IgM (,0.4 g/l). ^6,7^ - Presence of B cells but reduced memory B cell subsets and/ or increased CD21 low subsets by flow cytometry. ^8^ - IgG3 deficiency (<0.2 g/l). ^9,10^ - Impaired vaccine responses compared to age-matched controls. ^11^ - Transient vaccine responses compared with age-matched controls. ^12,13^ - Absent isohemagglutinins (if not blood group AB). ^14^ - Serological evidence of significant autoimmunity e.g. Coombes test - Sequence variations of genes predisposing to CVID e.g. *TACI, BAFFR, MSH5* etc. ^15,16^ |
| **D** | Presence of relatively specific histological markers of CVID (not required for diagnosis but presence increases diagnostic certainty, in the context of Category A and B criteria) |
|  | - Lymphoid interstitial pneumonitis. ^17^ - Granulomatous disorder. ^18,19^ - Nodular regenerative hyperplasia of the liver. ^20,21^ - Nodular lymphoid hyperplasia of the gut. ^22^ - Absence of plasma cells on gut biopsy. ^23,24^ |

Table 2. New Diagnostic criteria (Ameratunga et al 2013) for CVID. Meeting criteria in categories ABC or ABD indicates probable CVID. Patients meeting criteria ABC and ABD should be treated with IVIG/SCIG (Figure 1). Patients meeting criteria A alone, AB or AC or AD but not B, are termed possible CVID. Some of these patients may need to be treated with IVIG/SCIG. Patients with levels of IgG >5 g/l, not meeting any other criteria are termed hypogammaglobulinemia of uncertain significance (HGUS). ^1^ These diagnostic criteria must be applied sequentially as none are specific individually.


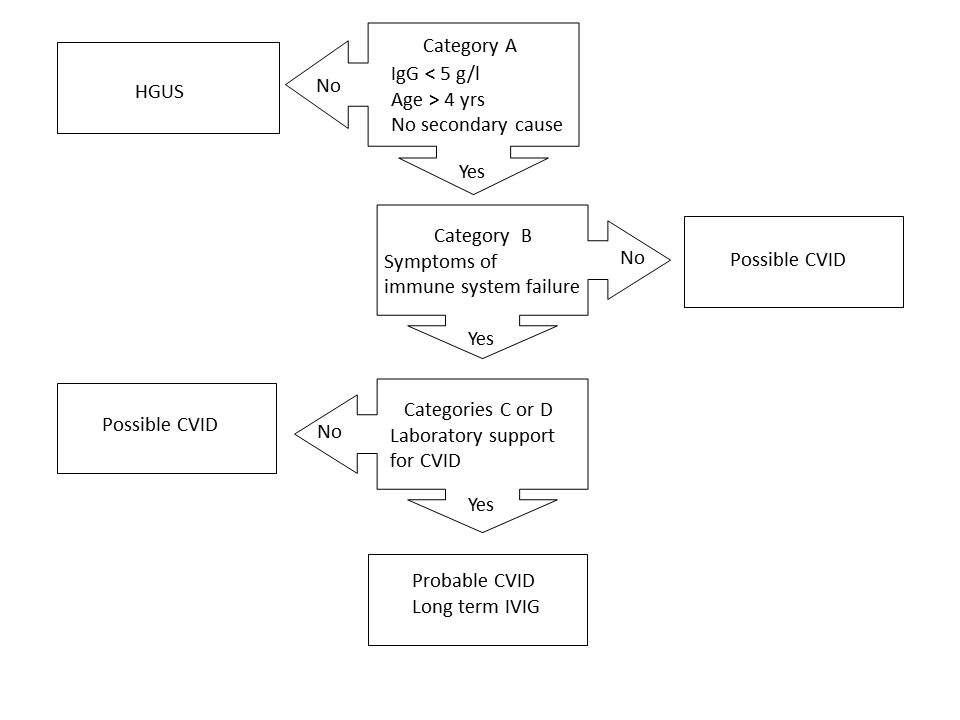


Figure 1. Treatment algorithm for CVID (Ameratunga et al 2013). ^1^ Patients must meet all major criteria in Category A for consideration of CVID. Category B confirms the presence of symptoms indicating immune system failure (ISF). To have probable CVID, patients must also have supportive laboratory evidence of immune system dysfunction (Category C) or characteristic histological lesions of CVID (category D). Patients with mild hypogammaglobulinemia (IgG >5 g/l) are termed hypogammaglobulinemia of uncertain significance (HGUS). Patients meeting Category A criteria but not other criteria are deemed to have possible CVID. Most patients with probable CVID are likely to require IVG/ SCIG. Some patients with possible CVID will require IVIG/ SCIG but most patients with HGUS are unlikely to need IVIG/ SCIG replacement.^1^

**ESID Registry criteria 2019^25^**

At least one of the following:

- increased susceptibility to infection
- autoimmune manifestations
- granulomatous disease
- unexplained polyclonal lymphoproliferation
- affected family member with antibody deficiency

AND marked decrease of IgG and marked decrease of IgA with or without low IgM levels

(measured at least twice; <2SD of the normal levels for their age)

AND at least one of the following:

- poor antibody response to vaccines (and/or absent isohemagglutinins); ie, absence of
- protective levels despite vaccination where defined
- low switched memory B cells (<70% of age-related normal value)

AND secondary causes of hypogammaglobulinemia have been excluded (eg, infection, protein

loss, medication, malignancy)

AND diagnosis is established after the fourth year of life (but symptoms may be present before)

AND no evidence of profound T-cell deficiency, defined as 2 of the following (y ¼ years of life):

- CD4 numbers/microliter: 2-6 y < 300, 6-12 y < 250, >12 y < 200
- % naïve of CD4: 2-6 y < 25%, 6-16 y < 20%, >16 y < 10%
- T-cell proliferation absent

**ESID Unclassified antibody deficiency (UCH)**

At least one of the following:

- Recurrent or severe bacterial infections
- Autoimmune phenomena (especially cytopenias)
- Polyclonal lymphoproliferation
- Affected family member

AND at least one of the following:

- marked decrease of at least one of total IgG, IgG1, IgG2, IgG3, IgA, or IgM levels
- failure of IgG antibody response(s) to vaccines

AND secondary causes of hypogammaglobulinemia have been excluded (eg, infection, protein

loss, medication, malignancy)

AND no clinical signs of T-cell related disease

AND does not fit any of the other working definitions (excluding “unclassified

immunodeficiencies”)

**Consensus definition of CVID (ICON) 2016^26^**

1. Most patients will have at least 1 of the characteristic clinical manifestations (infection, autoimmunity, lymphoproliferation). However, a diagnosis of CVID may be conferred on asymptomatic individuals who fulfill criteria 2 to 5, especially in familial cases.
2. Hypogammaglobulinemia should be defined according to the age-adjusted reference range for the laboratory in which the measurement is performed. The IgG level must be repeatedly low in at least 2 measurements more than 3 weeks apart in all patients. Repeated measurement may be omitted if the level is very low (<100–300 mg/dL depending on age), other characteristic features are present, and it is considered in the best interest of the patient to initiate therapy with IgG as quickly as possible
3. IgA or IgM level must also be low. (Note that some experts prefer a more narrow definition requiring low IgA level in all patients.)
4. It is strongly recommended that all patients with an IgG level of more than 100 mg/dL should be studied for responses to T-dependent (TD) and T-independent (TI) antigens, whenever possible. In all patients undergoing such testing, there must be a demonstrable impairment of response to at least 1 type of antigen (TD or TI). At the discretion of the practitioner, specific antibody measurement may be dispensed with if all other criteria are satisfied and if the delay incurred by prevaccination and postvaccination antibody measurement is thought to be deleterious to the patient’s health.
5. Other causes of hypogammaglobulinemia must be excluded (Table I).
6. Genetic studies to investigate monogenic forms of CVID or for disease-modifying polymorphisms are not generally required for diagnosis and management in most of the patients, especially those who present with infections only without immune dysregulation, autoimmunity, malignancy, or other complications. In these latter groups of patients, however, single gene defects may be amenable to specific therapies (eg, stem cell therapy) and molecular genetic diagnosis should be considered when possible.

1. Ameratunga R, Woon ST, Gillis D, Koopmans W, Steele R. New diagnostic criteria for common variable immune deficiency (CVID), which may assist with decisions to treat with intravenous or subcutaneous immunoglobulin. Clin Exp Immunol 2013;174:203-11.

2. Oksenhendler E, Gerard L, Fieschi C, et al. Infections in 252 patients with common variable immunodeficiency. Clin Infect Dis 2008;46:1547-54.

3. Agarwal S, Cunningham-Rundles C. Assessment and clinical interpretation of reduced IgG values. Ann Allergy Asthma Immunol 2007;99:281-3.

4. Chapel H, Cunningham-Rundles C. Update in understanding common variable immunodeficiency disorders (CVIDs) and the management of patients with these conditions. Br J Haematol 2009;145:709-27.

5. Knight AK, Cunningham-Rundles C. Inflammatory and autoimmune complications of common variable immune deficiency. Autoimmun Rev 2006;5:156-9.

6. Cunningham-Rundles C, Bodian C. Common variable immunodeficiency: clinical and immunological features of 248 patients. Clin Immunol 1999;92:34-48.

7. Chapel H, Lucas M, Lee M, et al. Common variable immunodeficiency disorders: division into distinct clinical phenotypes. Blood 2008;112:277-86.

8. Wehr C, Kivioja T, Schmitt C, et al. The EUROclass trial: defining subgroups in common variable immunodeficiency. Blood 2008;111:77-85.

9. Abrahamian F, Agrawal S, Gupta S. Immunological and clinical profile of adult patients with selective immunoglobulin subclass deficiency: response to intravenous immunoglobulin therapy. Clin Exp Immunol 2010;159:344-50.

10. Olinder-Nielsen AM, Granert C, Forsberg P, Friman V, Vietorisz A, Bjorkander J. Immunoglobulin prophylaxis in 350 adults with IgG subclass deficiency and recurrent respiratory tract infections: a long-term follow-up. Scand J Infect Dis 2007;39:44-50.

11. Musher DM, Manof SB, Liss C, et al. Safety and antibody response, including antibody persistence for 5 years, after primary vaccination or revaccination with pneumococcal polysaccharide vaccine in middle-aged and older adults. J Infect Dis 2010;201:516-24.

12. Koopmans W, Woon ST, Brooks AE, Dunbar PR, Browett P, Ameratunga R. Clinical variability of family members with the C104R mutation in transmembrane activator and calcium modulator and cyclophilin ligand interactor (TACI). J Clin Immunol 2013;33:68-73.

13. Grabenstein JD, Manoff SB. Pneumococcal polysaccharide 23-valent vaccine: long-term persistence of circulating antibody and immunogenicity and safety after revaccination in adults. Vaccine 2012;30:4435-44.

14. Tiller TL, Jr., Buckley RH. Transient hypogammaglobulinemia of infancy: review of the literature, clinical and immunologic features of 11 new cases, and long-term follow-up. J Pediatr 1978;92:347-53.

15. Pan-Hammarstrom Q, Salzer U, Du L, et al. Reexamining the role of TACI coding variants in common variable immunodeficiency and selective IgA deficiency. Nat Genet 2007;39:429-30.

16. Salzer U, Bacchelli C, Buckridge S, et al. Relevance of biallelic versus monoallelic TNFRSF13B mutations in distinguishing disease-causing from risk-increasing TNFRSF13B variants in antibody deficiency syndromes. Blood 2009;113:1967-76.

17. Popa V. Lymphocytic interstitial pneumonia of common variable immunodeficiency. Ann Allergy 1988;60:203-6.

18. Ameratunga R, Becroft DM, Hunter W. The simultaneous presentation of sarcoidosis and common variable immune deficiency. Pathology 2000;32:280-2.

19. Fasano MB, Sullivan KE, Sarpong SB, et al. Sarcoidosis and common variable immunodeficiency. Report of 8 cases and review of the literature. Medicine (Baltimore) 1996;75:251-61.

20. Fuss IJ, Friend J, Yang Z, et al. Nodular Regenerative Hyperplasia in Common Variable Immunodeficiency. J Clin Immunol 2013;33:748-58.

21. Malamut G, Ziol M, Suarez F, et al. Nodular regenerative hyperplasia: the main liver disease in patients with primary hypogammaglobulinemia and hepatic abnormalities. J Hepatol 2008;48:74-82.

22. Luzi G, Zullo A, Iebba F, et al. Duodenal pathology and clinical-immunological implications in common variable immunodeficiency patients. Am J Gastroenterol 2003;98:118-21.

23. Malamut G, Verkarre V, Suarez F, et al. The enteropathy associated with common variable immunodeficiency: the delineated frontiers with celiac disease. Am J Gastroenterol 2010;105:2262-75.

24. Agarwal S, Smereka P, Harpaz N, Cunningham-Rundles C, Mayer L. Characterization of immunologic defects in patients with common variable immunodeficiency (CVID) with intestinal disease. Inflamm Bowel Dis 2011;17:251-9.

25. Seidel MG, Kindle G, Gathmann B, et al. The European Society for Immunodeficiencies (ESID) Registry Working Definitions for the Clinical Diagnosis of Inborn Errors of Immunity. J Allergy Clin Immunol Pract 2019.

26. Bonilla FA, Barlan I, Chapel H, et al. International Consensus Document (ICON): Common Variable Immunodeficiency Disorders. J Allergy Clin Immunol Pract 2016;4:38-59.
